# Effectiveness of combined interventions to empower girls and address social norms in reducing child marriage in a rural sub-district of Bangladesh: A Cluster Randomised Controlled Trial of the Tipping Point Initiative

**DOI:** 10.7189/jogh.14.04020

**Published:** 2024-02-23

**Authors:** Ruchira Tabassum Naved, Sultan Mahmud, Mahfuz Al Mamun, Kausar Parvin, Sadhvi Kalra, Anne Laterra, Anne Sprinkel

**Affiliations:** 1 Maternal and Child Health Division, International Centre for Diarrhoeal Disease Research, Bangladesh (icddr,b), Dhaka, Bangladesh; 2 CARE USA, Atlanta, Georgia, USA

## Abstract

**Background:**

Elimination of girl child marriage (CM) globally at the current pace is projected to take about 300 years. Thus, innovative and effective solutions are urgently warranted. Bangladesh reports one of the highest rates of CM in the world. We present the impact of Tipping Point Initiative (TPI), a combined intervention to empower girls and to address social norms on CM in Bangladesh.

**Methods:**

A three-arm non-blinded Cluster Randomised Controlled Trial was conducted in 51 villages/clusters in a sub-district of Bangladesh. Clusters were randomly assigned to the arms: Tipping Point Program (TPP), Tipping Point Program Plus (TPP+), and Pure Control. TPP conducted 40 weekly single-gender group sessions with never-married adolescent girls and boys recruited at 12 −<16 years; and 18-monthly gender-segregated group sessions with the parents. On top of TPP, TPP+ included cross-gender and -generation dialogues, girls’ movement building and girl-led community sensitisation. Intention-to-treat analysis was performed to assess the impact of TPI on the hazard of CM, the primary outcome. The impact of girls’ session attendance on CM was also assessed. At baseline 1275 girls (TPP = 412; TPP+ = 420; Control = 443) were interviewed between February–April 2019. At endline 1123 girls (TPP = 363; TPP + = 366; Control = 394) were interviewed and included in the analyses.

**Results:**

No intervention impact was detected on the full sample (TPP vs. Control: adjusted hazard ratio (aHR) = 1.14; 95% CI = 0.79–1.63, *P* = 0.47), (TPP + vs. Control: aHR = 1.24; 95% CI = 0.89–1.71, *P* = 0.19, (TPP vs. TPP+: aHR = 1.03; 95% CI = 0.72–1.47, *P* = 0.87). However, in the TPP arm, the hazard of CM was reduced by 54% (aHR = 0.46; 95% CI = 0.23–0.92, *P* = 0.03) among the girls in the highest tertile of session attendance, compared to the lowest. In the TPP+ arm, this hazard was reduced by 49% (aHR = 0.51; 95% CI = 0.23–0.92, *P* = 0.03) among girls in the highest tertile, compared to the lowest tertile.

**Conclusions:**

Although TPI did not show an effect on CM in any of the intervention arms, within each intervention arm, a positive effect was detected in reducing CM among girls in the highest tertile of session attendance despite implementation challenges due to COVID-19.

**Registration:**

Clinicaltrials.gov: NCT03965273; Date: 29 May 2019.

Globally, 19% girls are married before 18 [1]. South Asia contributes to 29% of the global burden of girl child marriage (CM) with Bangladesh reporting the third highest prevalence of CM globally and the highest in South Asia [2]. Two in four women aged 20–24 reported being married before the age of 18 in Bangladesh [3]. There are, however, large geographical variations in the rate of CM here (23–64%) [3]. Poverty, low education, rural residence, pervasive patriarchal social norms, the dowry system, and the practice of linking family honour to girl’s sexuality are cited as important determinants of CM in Bangladesh [4,5]. Despite existing legal frameworks and programmes to address CM [6,7], the rate of CM reduction in Bangladesh was the slowest among the South Asian countries [8]. This is of particular concern, because even according to the current global trend in CM decline, elimination of CM is at least 300 years away [9].

To date, a variety of interventions have been implemented worldwide for addressing CM. Common intervention components include girls’ education/life skills, livelihoods/conditional cash transfer, empowerment, and community mobilisation [9]. While well-designed economic, education, and life skills interventions were found effective in reducing child marriage, girl-focused empowerment programmes presented mixed evidence of effectiveness [10,11]. Although it is imperative to change social, including gender norms driving CM for sustained elimination of the practice, there is, however, a dearth of interventions driven by sound theories of change and systematic attempts at changing the social norms facilitating CM [11]. As pointed out in the literature, lack of understanding of social norms, which social norms to target and how to change them effectively impede the development of effective and sustainable CM prevention programmes [10,12,13]. In Bangladesh, several rigorously evaluated CM interventions show promise. For instance, a Cluster Randomised Controlled Trial (CRCT) showed that financial incentives to delay marriage reduced the likelihood of CM by 21% (Unpublished material). A combination of financial incentives and empowerment or empowerment alone, however, did not show an effect on CM in Bangladesh (Unpublished material). Another CRCT conducted in southern Bangladesh reports that support in education, promotion of livelihood skills, and gender sensitisation interventions, each implemented separately, were effective in reducing CM [14]. Unfortunately, none of the effective interventions mentioned above extensively engaged with social norm change.

In this backdrop, CARE developed the Tipping Point Initiative (TPI) aimed to empower adolescent girls and change social norm for reducing CM. The first phase of TPI was deemed successful in identifying the prominent latent norms that perpetuate CM. When developing the intervention design for Phase 2, these norms were targeted to reduce rates of child marriages [15,16]. In line with this, TPI phase 2 focused on building adolescent girls’ agency, creating supporting relations and transforming norms that drive CM. The intervention targeted social norms around girls’ mobility; decision-making regarding own marriage; interaction with boys; riding bicycle and playing male sports; and collective action for rights. This paper presents the findings from the impact evaluation of TPI phase 2 in reducing CM.

## METHODS

### Study design

The TPI phase 2 evaluation employed a non-blinded three-arm Cluster Randomised Controlled Trial (CRCT) design. The arms were as follows: (a) Tipping Point Program (TPP): designed to enhance adolescent girls’ personal assets, intrinsic, and instrumental agency; (b) Tipping Point Program Plus (TPP+): TPP intervention with additional elements designed to enhance social norms change by engaging community leaders and facilitating girl-led community activities; and (c) Pure control. This design allowed us to assess the effectiveness of: a) TPP+ intervention; b) TPP intervention; and c) TPP+ intervention over TPP intervention in reducing CM. The detailed methodology has been presented elsewhere [17]. The study received ethical approval from icddr,b’s Institutional Review Board (PR#18056). The trial was registered with the ClinicalTrials.gov registry, NCT03965273.

### Study site

TPI phase 2 was implemented in 51 villages/clusters, in purposively selected Pirgacha, a sub-district in Bangladesh under Rangpur division characterised by high level of poverty [18] and the lowest median age at first marriage (15.7 years) in the country [19].

Villages were considered as clusters in this study and the following strategies were employed in selecting them. First of all, a village was randomly selected from a comprehensive list of villages in Pirgacha. Then, the subsequent village was selected allowing for a ‘buffer zone’. In this study, a buffer zone refers to the village/s sharing a border with any selected village/cluster and separating a selected cluster from another. Buffer zones were allowed to avoid intervention contamination from intervention to control clusters. This process was continued until 51 villages were chosen and then each selected village was randomised (1:1:1) into one of the three study arms (17 per arm) by a statistician from icddr,b using a computer-generated sequence.

### Sample size

Fifty-one clusters (17 per arm) were required to detect a 15% reduction in CM considering cluster size of 22, intra-cluster correlation of 0.05, 5% significance level and 80% power. Allowing for a 15% non-response/lost to follow-up the cluster size increased to 25 and total sample size reached to 1275 girls. A sample size of 540 community members was required to detect an increase of 15% in positive social norms around CM with 5% significance level, 80% power and 5% non-response rate. To ensure the participation of both women and men we required six men and six women from each cluster.

### Participants

Girls aged 12−<16 years, never-married, and usual residents of the study villages were eligible to enrol in the study. Simple random samples of 29 eligible girls were drawn from the list of eligible girls in each cluster derived from household enumeration conducted during January–March 2019. Considering possible refusal, we oversampled the girls by 16% to achieve a group size of 25. In clusters with ≤29 eligible girls, all were included in the list. For the community survey, two different cross-sectional samples of six women and six men villagers aged ≥25 years were randomly selected from each cluster and interviewed at baseline and endline surveys. Eligible girls and community members were selected randomly using computer generated sequence by the same statistician who performed the village randomisation, but did not take part in the recruitment of programme/study participants.

### Blinding

No one was blinded in this study.

### The Tipping Point Intervention

**Figure 1:**
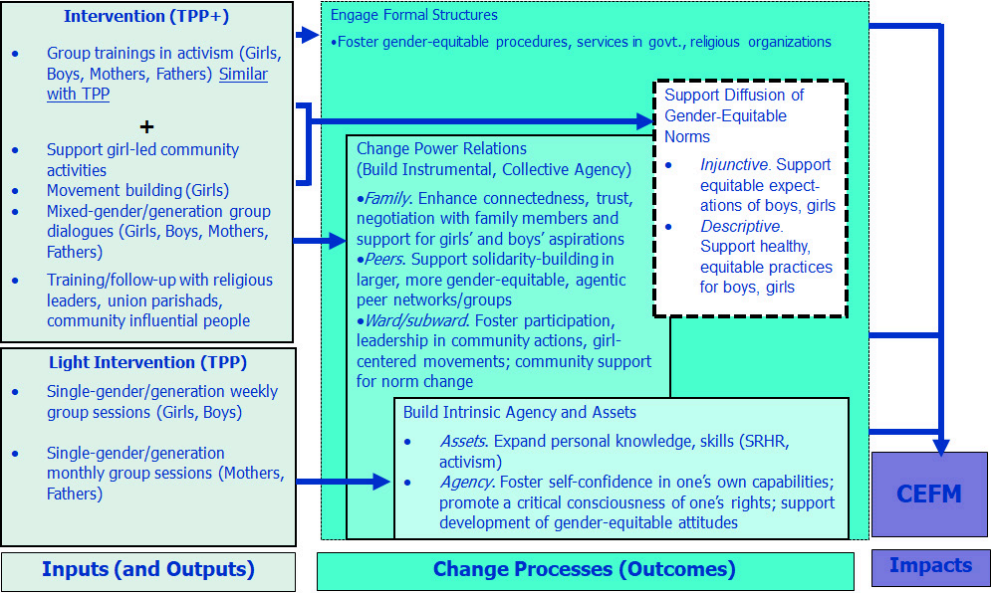
The Tipping Point Theory of Change. SRHR – Sexual and Reproductive Health and Rights, TPP – Tipping Point Program, TPP+ – Tipping Point Program Plus

TPI aspired to empower girls and address social norms that restrict the lives and roles of girls and uphold the practice of CM. The approach focused on synchronised engagement with different participant groups to promote the rights of adolescent girls through community-level programming. TPI developed two implementation packages, Tipping Point Program (TPP) and Tipping Point Program Plus (TPP+), following a Theory of Change (ToC) (**Figure 1**) based on a multi-year phase of formative research, exploration, and community-action research to ensure that the packages were well-tailored to address the root causes of CM in these specific communities. The resulting approaches were rooted in challenging social expectations and repressive norms and promoting girl-driven movement-building and activism; components designed to help adolescent girls to find and collectively step into spaces to engage with and tackle inequality.

The TPI ToC was focused on three domains of change: (1) the individual agency that builds consciousness, confidence, self-esteem, and aspirations and empowers with knowledge, skills and capabilities; (2) the power relations through which people, particularly women and girls, navigate their lives; and (3) the structures that inhibit or promote equitable access to services and protection from harm. The inputs of the Tipping Point Initiative (**Figure 1**) illustrate the project components in both the core model, TPP, and the enhanced model, TPP+. The Initiative posits that these components will impact each of these domains in turn. At an individual level, through group sessions and dialogues, the Initiative will build agency and assets of the girls. Working with girls’ family members, peers, and community members will encourage more trusting relationships with girls resulting into support of their aspirations. The TPP+ model will work with formal structures (e.g. schools, religious institutions, health care providers) to address systemic barriers that girls and their family members face in efforts to realise their full potential through awareness raising with stakeholders and girl-led collective action. Both models also include activities designed to make visible and diffuse equitable, healthy norms and behaviours for girls and boys that each of these components makes possible. Together, these changes are expected to contribute to reductions in CM.

**Table 1** presents detailed information on intervention implementation. Both the intervention packages included a core set of interventions (group sessions with adolescent girls (TPP = 412, TPP+ = 420) and boys (TPP = 408, TPP+ = 418) aged 12 −<16 years, and mothers (TPP = 421, TPP+ = 404) and fathers (TPP = 414, TPP+ = 418) of adolescent girls and boys. The key components of the sessions included:

**Table 1:** The Tipping Point Initiative as implemented

Participants’ groups		Sessions/activities	Core sessions/trainings
Core participants’ groups	Adolescent girls	40 weekly sessions (45 sessions in original plan)	Social norms (all participant groups): equity and equality; rights and duties; gender; patriarchy; power and privileges; puberty; sex and love; honour; GBV; child marriage. Access to alternatives (girls' groups only): financial literacy and girls from the group who are interested participate in Village Savings and Loan Association (VSLA) (Starting in the 7th month). ASRHR (all participants' group): menstruation; masculinities; female sexuality; contraception; HIV/AIDs. Girls-centred movement building (girls’ group only): (starting in the 7th month): leadership; empowerment dialogues; collective action; civic participation.
	Adolescent boys	40 sessions (45 sessions in original plan)	
	Mothers group	18 monthly sessions	
	Fathers group	18 monthly sessions	
Other participants*	Religious leaders	As needed	Intensive trainings, follow-up meetings
	Local government (union parishads)		
	Influential people		
Activist training*	Selected champion boys, fathers, mothers	As needed	Trainings and meetings to support adolescent girls’ activism: starting in the 7th month.
	Selected girl leaders	4 community level activities	Girl leaders receive training on campaigning and activism, linked to other girls’ groups & networks, and given access to a budget and mentorship to execute 4 community level activities.
Girl-led activities*	Community members	4 community level social norms activities (6 activities in original plan)	Organised and led by adolescent girls’ groups on following themes: mobility, menstruation, gender division of labour, dowry, family honour/sexual harassment, girls’ aspirations
	Community members	3 activist-led activities (4 activities in original plan)	Created, organised and led by network of activist girls: the network of girl leaders elected across villages organised and executed 4 activities of their own choice in each of their communities, using their own budget.
Joint sessions*	Adolescent girls and boys, and their mothers and fathers	4 inter-group dialogues (6 dialogues in original plan)	Facilitated dialogues between core participants groups in the following combinations: 1) adolescent girls with boys, 2) adolescent girls with mothers, 3) mothers with fathers, 4) adolescent girls, adolescent boys, mothers, and fathers.

TPP – Tipping Point Program, TPP+ – Tipping Point Program Plus

*Indicates components that are part of the TPP+ and are not present in the TPP.

- social norms (all participant groups): child rights, gender and sexuality, patriarchy, power and privileges, puberty, sex and love, honour, dowry, gender division of labour, gender-based violence, child early and forced marriage (CEFM).

- access to alternatives (girls’ groups only): financial literacy and an opportunity to join a Village Savings and Loans Association (VSLA).

- adolescent Sexual and Reproductive Health and Rights (ASRHR) (all participants’ groups): sexual and reproductive rights; menstruation; masculinities; female sexuality; contraception; HIV/AIDS.

- girl-centred movement building (girls’ groups only): leadership; empowerment dialogues; collective action; civic participation.

In addition, TPP+ included the following set of emphasised social norms change activities:

- intergroup dialogues: held between core participant groups, i.e. girls with boys, girls with mothers, mothers with fathers, and finally girls with boys, mothers, and fathers.

- girls’ activist training (selected girl leaders): girl leaders received training on campaigning and activism, were linked to other girls’ groups and networks, and had access to a budget and mentorship to execute community-level activities.

- activist training for allies (selected champion boys, fathers, and mothers): trainings and meetings to support adolescent girls’ activism.

- other participant groups: religious leaders, local government, and other community-level influencers were engaged in quarterly discussion and dialogues

- girl-led activities: organised and led by adolescent girls’ groups on mobility, menstruation, dowry, gendered division of labour, family honour, sexual harassment, and girls’ aspirations

- community activities: the network of girl leaders elected across villages organised and executed activities of their choice in each of their communities, using a budget.

Gram Bikash Kendra (GBK), a local non-government organisation, delivered the intervention. Due to COVID-19, the planned 18-month intervention ended up being a 17-month intervention implemented over a 20-month period (April 2019 – December 2020). The number of sessions with the girls was reduced from 45 to 40 by merging a few sessions. During the lockdown face-to-face session delivery was replaced by virtual sessions. Similar strategies were adopted for sessions with the boys and parents. The number of community-wide activities were reduced from ten to seven. Four cross-gender and/or generational dialogues were held instead of six.

### Survey data collection

Baseline data were collected during February-April 2019 and endline data were collected during November–December 2021. The questionnaires for girls’ and community surveys were pre-tested and piloted before the baseline surveys (available at: https://osf.io/nhdsc/). Twenty interviews were conducted for pre-testing the girls’ questionnaire, while 10 female and 10 male community members were interviewed for pre-testing the community questionnaire. During the piloting, 30 interviews with girls; and 15 interviews with females and 15 interviews with male community members were conducted. The questionnaires were finalised incorporating all the feedback received from pre-testing and piloting. Data were collected in Bangla using face-to-face interviews. The oral assent of adolescent girls and the oral consent of their parents for the girls’ survey. Oral consent was obtained from the participants in the community survey. Gender-matched interviewers conducted the interviews using Tablets. The interviews were conducted in private at a location convenient for the participants. A survey team of 14 female data collectors, two male data collectors, four supervisors, two quality control officers (QCOs) and one survey coordinator were employed for baseline and endline girls’ and community surveys. Before baseline and endline, the survey teams received a 12-day participatory training on gender, child marriage, and empowerment of adolescent girls, research ethics, survey methods, the questionnaire, and the use of tablets for error-free data entry.

### Data quality monitoring

A comprehensive data quality monitoring system was in place. The supervisors observed the quality of the interviews. Daily team meetings were held by the survey team. Five percent of the study participants were revisited by the supervisors. They administered a short questionnaire focused mainly on adherence to ethical guidelines and the administration of questions on particular topics. Each completed questionnaire was rechecked by the QCO on a daily basis. Further, a computer-based data checking routine was used weekly to detect any inconsistencies in the data.

### Outcomes and measurement

TPI included one primary and 12 secondary outcomes (**Table 2**).

**Table 2:** Measurement of Tipping Point Initiative primary and secondary outcomes

Serial	Outcomes	Questions/scale used	Number of items	Cronbach’s Alpha	Kaiser–Meyer–Olkin (KMO)	Expected direction of change
**Primary outcome**						
Hazard of girl child marriage		Marital status and age at first marriage	–	–	–	Decrease
**Secondary outcomes**						
Girls’ self-efficacy		Girls’ perceived confidence in achieving life goals in education, health care, mobility, marriage, and income earning	8	0.79	0.80	Increase
Collective efficacy		Questions were framed around collective action involving the community around preventing child marriage, preventing violence against girls, etc.	4	0.83	0.79	Increase
Girls’ knowledge regarding Sexual and Reproductive Health and Rights (SRHR)		Questions were framed around their knowledge about SRHR	–	–	–	Increase
Girls’ positive attitudes regarding gender roles		Modified version of the Gender-Equitable Men (GEM) Scale [20].	7	0.70	0.81	Increase
Girls’ endorsement of control of adolescent girls by family members		Modified version of the Gender-Equitable Men (GEM) Scale [20].	4	0.76	0.73	Decrease
Girls’ endorsement of justification of girl-beating		Modified version of the Gender-Equitable Men (GEM) Scale [20]. Three sub-scales were constructed for girls	8	0.78	0.82	Decrease
Girls’ cohesion		The neighbourhood cohesion scale [21]	13	0.93	0.95	Increase
Girls’ confidence in negotiation skills		Three questions were asked to measure confidence in negotiating education, marriage, and mobility	3	0.72	0.68	Increase
Girls’ mobility		Questions were framed around girl’s ability to move certain places	6	0.53	0.69	Increase
Girls’ participation in financial activities		Six questions related to their involvement in financial activities were formulated	–	–	–	Increase
Girls’ connectedness with parents		Connectedness was measured by asking several questions about their relations with parents	7	0.77	0.82	Increase
Community positive social norm (community members’ reporting)		Measured using statements about normative expectations (injunctive norms) regarding girls’ practices and parents’ practices	8	0.71	0.80	Increase

#### Primary outcome: The hazard of child marriage (CM)

The outcome of our interest was the hazard of CM, where any marriage of girls under 18 years was considered as CM. The dependent variable was the time to first marriage calculated from data on marital status (‘1’ if married, and ‘0’ otherwise) and age at first marriage (in years).

#### Secondary outcomes

All the secondary outcome variables were nonnegative continuous variables. Factor analyses were performed to validate all the scales used in the analysis (**Table 2**). Where necessary, the items in the scales were recoded so that all were anchored at 0. Summative scores were obtained for each scale. The scores were then divided into tertiles for use in the models as independent variables.

Girls' self-efficacy was evaluated based on their confidence in achieving life goals in education, health care, mobility, marriage, and income earning. Girls' collective efficacy, on the other hand, explored their beliefs in undertaking collective action to prevent child marriage and violence against girls. The study also assessed girls' knowledge regarding sexual and reproductive health and rights (SRHR), using questions on menstruation, reproductive health, contraceptives, and sexually transmitted diseases. Girls' attitudes toward gender roles, control of girls by the family members, and justification of girl-beating were measured using a modified version of the Gender-Equitable Men (GEM) Scale [20]. Girls' cohesion was examined using the neighbourhood cohesion scale [21]. We also measured girls' confidence in negotiating education, marriage, and mobility with their parents; their participation in financial activities and financial decision-making; and their connectedness with parents. The community sample was used to measure positive social norms regarding girls' mobility, riding and playing, collective action for girls' rights, and girls’ participation in decision-making regarding own marriage. The details regarding the measurement of each secondary outcome have been presented elsewhere [17].

### Covariates

The definition and measurement/coding of covariates included in the analyses are presented in the **Online Supplementary Document**. The covariates included in the regression analyses are listed below. The individual level covariates included girls’ age, education, religion, ownership of asset, and group membership. The household level covariates were household wealth index, and household head’s education. The village level covariates used were women’s education in the village and religious composition. In the analysis of the impact of TPI on social norms, considered as very important secondary outcomes we have used data from the community survey. In the models run for the purpose we have controlled for community members age, education, marital status, and religion.

### Statistical analysis

The effects of TPI were determined by comparing the three arms as follows: (1) TPP – Pure control = effect of TPP intervention; (2) TPP+ – Pure control = effect of TPP+ intervention; (3) TPP+ – TPP = effect of emphasised social norms change. Intention-to-treat analysis was used to measure the impact of TPI on the primary outcome, i.e. the hazard of CM. Multilevel parametric survival models (multilevel inverse-Gaussian frailty model) [22,23] were fitted. In addition, we explored the dose-response effect of the number of sessions attended by the girls in each intervention arm on the hazard of child marriage. For this purpose, we have calculated and used in the analysis tertiles of session attendance in each intervention arm. Cluster-level proportions of session attendance by fathers were also included in the survival models.

We tested the normality of the secondary outcome variables using the Shapiro-Wilk normality test and found that all these variables had a non-normal distribution. Therefore, we measured the impact of TPI on the secondary outcomes using generalised linear model with gamma distribution and log link function [24] adjusting for the baseline values. There was no specific data monitoring committee, however, there was one dedicated researcher responsible for ensuring data quality. All statistical analyses were performed using Stata v. 16.0 software (Stata Corporation LLC, College Station, TX, USA). The significance level was set at *P* = 0.05.

## RESULTS

**Figure 2:**
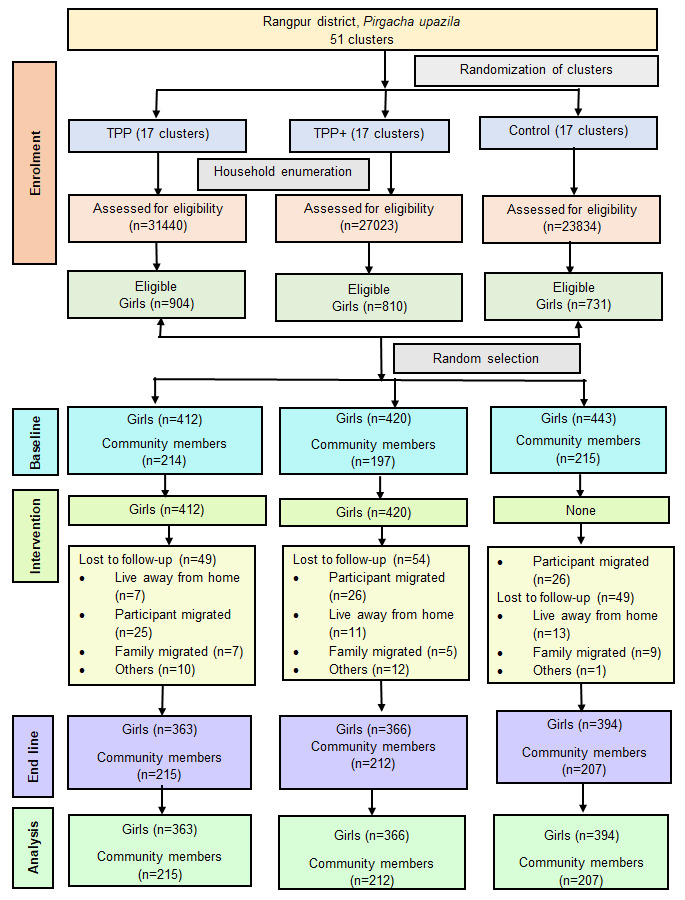
Trial profile of the study. TPP – Tipping Point Program, TPP+ – Tipping Point Program Plus

**Figure 2** presents the trial profile of the study. The study participants were recruited during February–April 2019. According to the household enumeration data, a total of 2445 girls were eligible to participate in the intervention. Of them, 1479 girls were randomly selected, and 1275 girls (TPP = 412, TPP+ = 420, control = 443) were finally interviewed at the baseline after obtaining consent from their guardians and assent from them. Of them, 1123 girls were interviewed at endline, with a retention rate of 88%. The main reasons for dropout (152) included absence from household (129 (85%)) mainly due to out-migration related to marriage, employment, and out-migration of the whole household. A significantly higher proportion of the girls dropped out from the lowest wealth quintile compared to the other wealth quintiles (data not shown), hence, the results were wealth adjusted. All girls interviewed successfully at both baseline and endline (n = 1123) were included in the impact evaluation analyses. Data from two different cross-sectional samples of community members from baseline (n = 626) and endline (n = 634) were also analysed. Though the target sample size was the same in both rounds, the final sample size achieved were different due to different non-response rates in different rounds.

At baseline, the girls were aged around 13 years on average across arms (**Table 3**). The mean years of education among girls was around seven years in all arms with most having six to seven years of education. The girls were predominantly Muslims across arms. Most of the girls were from the second highest wealth quintile in control and TPP arm and from the middle wealth quintile in the TPP+ arm.

**Table 3:** Baseline characteristics of the intention-to-treat sample

Characteristics	Control (n = 394)	TPP (n = 363)	TPP+ (n = 366)	Full sample (n = 1123)
Age in y, mean (SD)	13.50 (1.08)	13.50 (1.09)	13.60 (1.08)	13.50 (1.08)
Education in y, mean (SD)	6.80 (1.55)	6.60 (1.50)	6.70 (1.50)	6.70 (1.53)
Religion, n (%)				
*Muslim*	343 (87)	341 (94)	341 (93)	1025 (91)
*Hindu*	51 (13)	22 (6)	25 (7)	98 (9)
Wealth index, n (%)				
*Lowest*	79 (20)	71 (20)	76 (21)	226 (20)
*Second*	85 (22)	78 (21)	71 (19)	234 (21)
*Middle*	78 (20)	51 (14)	85 (23)	214 (19)
*Fourth*	87 (22)	82 (23)	79 (22)	248 (22)
*Highest*	65 (17)	81 (22)	55 (15)	201 (18)

SD – standard deviation, TPP – Tipping Point Program, TPP+ – Tipping Point Program Plus

The girls attended on average 28 and 29 group sessions in the TPP and TPP+ arms, respectively (**Table 4**). In the TPP arm, 37% of the girls were in the lowest tertile and attended 0–27 sessions; 33% were in the middle tertile and attended 28–34 sessions; while 30% of the girls were in the highest tertile and attended 36–40 sessions. None of them received 35 group sessions. In the TPP+ arm, 33% of the girls were included in the lowest tertile and they attended zero to 26 sessions. About 34% of the girls were in the middle tertile and attended 27–37 sessions, while 32% of the girls were in the highest tertile and attended 39–40 sessions. None of them received 38 group sessions. No significant difference was detected in the baseline characteristics, except religion, of the girls from different tertiles in both intervention arms (Table S1 in **Online Supplementary Document**). However, religion was controlled in the models.

**Table 4:** Girls’ session attendance

Characteristics	Mean (SD)	n (%)
**TPP arm**		363 (100)
Girls’ session attendance	28 (11)	
Girls’ session attendance, tertiles		
*Lowest (0–27)*		136 (37)
*Middle (28–34)*		119 (33)
*Highest (36–40)*		108 (30)
**TPP+ arm**		366 (100)
Girls’ session attendance	29 (12)	
Girls’ session attendance, tertiles		
*Lowest (0–26)*		122 (33)
*Middle (27–37)*		126 (34)
*Highest (39–40)*		118 (32)

SD – standard deviation, TPP – Tipping Point Program, TPP+ – Tipping Point Program Plus

**Figure 3:**
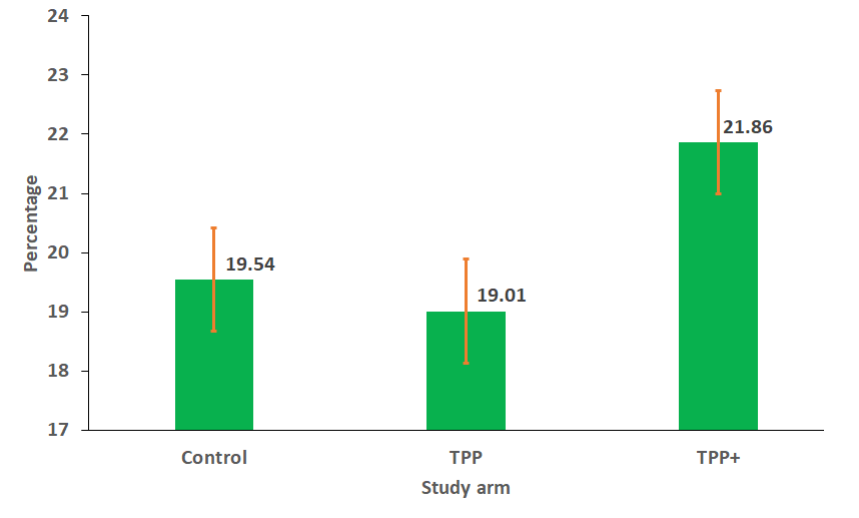
Prevalence of child marriage among Tipping Point adolescent girls at endline.

At endline, 20% (77 / 394) of the girls aged 14–18 were married before 18 in the control arm, 19% (69 / 363) in TPP and 22% (80 / 366) in TPP+ arms (**Figure 3**). The results of multilevel parametric survival analyses show no significant impact of any of the interventions on the hazard of CM (**Table 5**). However, an analysis of the impact of girls' session attendance on CM showed that in the TPP arm, the hazard of child marriage (CM) was reduced by 54% (adjusted hazard ratio (aHR) = 0.46; 95% CI = 0.23–0.92, *P* = 0.03) for the girls in the highest tertile who attended 36–40 group sessions, compared to those in the lowest tertile who attended zero to 27 group sessions (**Table 6**). In the TPP+ arm, the hazard of CM was reduced by 49% (aHR = 0.51; 95% CI = 0.23–0.92, *P* = 0.03) among girls in the highest tertile who attended 39 to 40 group sessions, compared to those in the lowest tertile who attended zero to 26 group sessions.

**Table 5:** Impact of Tipping Point Initiative on child marriage

Intervention	Effect* (95% CI)	*P-*value
Control arm (n = 394) (ref)	–	
TPP arm (n = 363)	1.14† (0.79–1.63)	0.46
TPP+ arm (n = 366)	1.24‡ (0.89–1.71)	0.19
Emphasised social norms component	1.03§ (0.72–1.47)	0.86

CI – confidence interval, ref– reference category, TPP – Tipping Point Program, TPP+ – Tipping Point Program Plus

*Adjusted hazard ratio. Results are from multilevel parametric survival models (multilevel inverse-Gaussian frailty models). Effect sizes are adjusted for covariates at two different levels. Individual/household level covariates: girls’ age, education, religion, household wealth index, and household head’s education. Village level covariates: women’s education, religion.

†Hazard ratio corresponding to TPP and control arms.

‡Hazard ratio corresponding to TPP+ and control arms.

§Hazard ratio corresponding to TPP+ and TPP arms.

**Table 6:** Impact of Tipping Point Initiative on child marriage by session attendance

Characteristics	Effect* (95% CI)	*P-*value
Girls’ session attendance in TPP arm		
*Lowest (ref.)*	–	
*Middle*	0.92 (0.53–1.61)	0.78
*Highest*	0.46 (0.23–0.92)	0.03
Girls’ session attendance in TPP+ arm		
*Lowest (ref.)*	–	
*Middle*	0.83 (0.49–1.04)	0.49
*Highest*	0.51 (0.29–0.94)	0.03

CI – confidence interval, ref– reference category, TPP – Tipping Point Program, TPP+ – Tipping Point Program Plus

*Adjusted hazard ratio. Results are from multilevel parametric survival models (multilevel inverse-Gaussian frailty models). Effect sizes are adjusted for covariates at two different levels. Individual/household level covariates: girls’ age, education, religion, household wealth index, and household head’s education. Village level covariates: women’s education, religion.

Further, we have analysed the impact of TPI session attendance on the secondary outcomes in relation to girls (**Table 7**). In the TPP arm, girls' self-efficacy increased by 6% (β = 0.06; 95% CI = 0.004–0.11, *P* = 0.03) among those who belong to the highest tertile of session attendance (36–40 sessions) compared to those in the lowest tertile (0–27 sessions). The effect size was the same on girls' self-efficacy in the TPP+ arm (β = 0.06; 95% CI = 0.01–0.11, *P* = 0.02) for the highest tertile (39–40 sessions) compared to those in the lowest tertile (0–26 sessions). In the TPP+ arm, a 6% increase was also detected in girls’ confidence in negotiation skills (β = 0.06; 95% CI = 0.13–0.11, *P* = 0.01) in the middle tertile (27–37 sessions) and by 8% (β = 0.08; 95% CI = 0.04–0.13, *P* = 0.00) in the highest tertile (39–40 sessions) compared to the lowest tertile (0–26 sessions). In this arm, girls' endorsement of the justification of girl-beating was reduced by 17% (β = −0.19; 95% CI = −0.37, −0.002, *P* = 0.04) for those in the highest tertile (39–40 sessions) compared to those in the lowest tertile (0–26 sessions). Moreover, girls' cohesion increased by 4% (β = 0.04; 95% CI = 0.009–0.07, *P* = 0.01) in the highest tertile (39–40 sessions) compared to the lowest tertile (0–26 sessions). The collective efficacy of girls also increased in this arm by 4% (β = 0.04; 95% CI = 0.002–0.08, *P* = 0.03) for those in the middle tertile (27–37 sessions) and by 5% (β = 0.05; 95% CI = 0.009–0.08, *P* = 0.01) for those in the highest tertile (39–40 sessions) compared to those in the lowest tertile (0–26 sessions).

**Table 7:** Impact of Tipping Point Program Plus on secondary outcomes by session attendance

	TPP			TTP+		
**Outcomes**	Effect* (95% CI)	***P-*value**	**% change†**	Effect* (95% CI)	***P-*value**	**% change†**
Sexual and reproductive health knowledge by session attendance‡						
*Lowest (ref.)*	–		–			
*Middle*	0.05 (−0.17, 0.06)	0.38	5	0.05 (−0.07, 0.17)	0.42	5
*Highest*	0.09 (−0.21, 0.04)	0.16	9	0.03 (−0.09, 0.16)	0.59	3
Positive attitudes regarding gender roles by session attendance§						
*Lowest (ref.)*	–		–			
*Middle*	−0.02 (−0.13, 0.09)	0.75	−2	−0.10 (−0.02, 0.22)	0.09	−10
*Highest*	−0.09 (−0.21, 0.02)	0.09	−9	−0.11 (−0.02, 0.22)	0.09	−11
Endorsement family members’ control over girls by session attendance§						
*Lowest (ref.)*	–		–			
*Middle*	−0.02 (−0.10, 0.06)	0.58	−2	−0.01 (−0.09, 0.07)	0.81	−1
*Highest*	0.02 (−0.06, 0.11)	0.57	2	0.03 (−0.05, 0.12)	0.46	3
Endorsement of justification of girl-beating by session attendance§						
*Lowest (ref.)*	–		–			
*Middle*	0.05 (−0.12, 0.22)	0.60	5	−0.16 (−0.34, 0.03)	0.09	−15
*Highest*	0.03 (0.14–0.21)	0.71	3	−0.19 (−0.37, −0.002)	0.04	−17
Confidence in negotiation by session attendance‡						
*Lowest (ref.)*	–		–			
*Middle*	−0.02 (−0.06, 0.03)	0.41	−2	0.06 (0.13–0.11)	0.01	6
*Highest*	0.002 (−0.05, 0.05)	0.93	0.2	0.08 (0.04–0.13)	0.00	8
Self-efficacy by session attendance‡						
*Lowest (ref.)*	–		–			
*Middle*	−0.01 (−0.04, 0.06)	0.82	−1	0.05 (−0.01, 0.09)	0.07	−5
*Highest*	0.06 (0.004–0.11)	0.03	6	0.06 (0.01–0.11)	0.02	6
Girls’ mobility by session attendance‡						
*Lowest (ref.)*	–		–			
*Middle*	−0.05 (−0.14, 0.03)	0.22	−5	0.02 (−0.07, 0.11)	0.71	2
*Highest*	−0.07 (−0.16, 0.02)	0.11	−7	0.06 (−0.03, 0.15)	0.21	6
Participation in financial activities and decision-making by session attendance‡						
*Lowest (ref.)*	–		–			
*Middle*	0.02 (−0.06, 0.24)	0.27	2	−0.02 (−0.16, 0.13)	0.78	2
*Highest*	−0.05 (−0.21, 0.11)	0.54	−5	−0.02 (−0.17, 0.13)	0.76	−2
Girls’ cohesion by session attendance‡						
*Lowest (ref.)*	–		–			
*Middle*	0.01 (−0.02, 0.05)	0.40	1	0.03 (−0.002, 0.06)	0.06	3
*Highest*	0.02 (−0.003, 0.06)	0.07	2	0.04 (0.009–0.07)	0.01	4
Collective efficacy by session attendance‡						
*Lowest (ref.)*	–		–			
*Middle*	0.03 (−0.01, 0.07)	0.16	3	0.04 (0.002–0.08)	0.03	4
*Highest*	0.04 (−0.007, 0.08)	0.10	4	0.05 (0.009–0.08)	0.01	5
Connectedness with parents by session attendance‡						
*Lowest (ref.)*	–		–			
*Middle*	−0.03 (−0.05, 0.003)	0.09	−3	−0.008 (−0.03, 0.02)	0.57	−0.8
*Highest*	−0.003 (−0.03, 0.03)	0.84	−0.3	0.01 (−0.02, 0.04)	0.37	1

CI – confidence interval, ref– reference category, TPP – Tipping Point Program, TPP+ – Tipping Point Program Plus

*Adjusted regression coefficient. Results are from generalised linear models with gamma distribution and log link function.

†% change calculated as (exp(*β)-*1)*100.

‡The models were adjusted for covariates: girls’ education, religion, ownership of asset, group membership, and household’s wealth index.

§The models were adjusted for covariates: girls’ education, religion, and household’s wealth index.

Findings from the generalised linear regression analysis of community-level secondary outcome (social norm) using community survey data (**Table 8**) show that the emphasised social norms component comprising of community sensitisation and girls’ movement building positively changed social norm around girls’ participation in decision making regarding own marriage by 27% (β = 0.24; 95% CI = 0.03–0.45, *P* = 0.02).

**Table 8:** Impact of Tipping Point Initiative on social norms, community adults report

Intervention	Social norm around girls’ mobility in and around the village			Social norm around girls’ riding and playing in the village			Social norm around girls’ decision making regarding own marriage			Social norm around girls’ collective action for girls’ rights		
	Effect* (95% CI)	*P*-value	% change†	Effect* (95% CI)	*P*-value	% change†	Effect* (95% CI)	*P*-value	% change†	Effect* (95% CI)	*P*-value	% change†
TPP+ ((TPP+)-control)	0.41 (−0.06, 0.89)	0.09	51%	0.14 (−0.21, 0.50)	0.42	15%	0.14 (−0.06, 0.34)	0.16	15%	0.04 (−0.14, 0.21)	0.68	4%
Emphasised social norms component ((TPP+)-TPP)	−0.03 (−0.54, 0.49)	0.92	−3%	−0.12 (−0.51, 0.26)	0.52	−11%	0.24 (0.03, 0.45)	0.02	27%	−0.04 (−.22, 0.14)	0.66	−4%

CI – confidence interval, ref– reference category, TPP – Tipping Point Program, TPP+ – Tipping Point Program Plus

*Adjusted regression coefficient. The models were adjusted for covariates: community members age, education, marital status, religion. Higher scores indicate more positive social norm. Results are from generalised linear models with gamma distribution and log link function.

†% change calculated as (exp(*β)-*1)*100.

## DISCUSSION

Overall, TPI did not show an effect on CM in any of the intervention arms – TPP or TPP+. However, within each intervention arm a positive effect of TPI was detected in the highest tertile of session attendance. Thus, compared to the lowest tertile of session attendance the hazard of CM was reduced in the highest tertile by 54% in TPP and 49% in TPP+ arms. Globally, reducing CM poses a great challenge to the policymakers, programme developers, and implementers. A recent estimate shows that at the current pace, the elimination of CM globally will take 300 years [9]. Within South Asia [3], Bangladesh has typically demonstrated the slowest pace in CM reduction, which raises legitimate concern regarding the achievement of SDG target for eliminating CM by 2030. The large positive effect of TPI in reducing CM among girls who received the highest dose of the intervention in each intervention arm is thus of particular importance. It is noteworthy that such effect has not been shown in any previous rigorously studied interventions in Bangladesh or elsewhere [25].

The study by Buchman et al [6] achieved a 25% reduction in CM in rural Bangladesh using conditional incentive intervention to the parents not to marry off the girls before 18 years of age. The Balika study [14] in southern Bangladesh showed a 23% reduction in child marriage as a result of a livelihood programme; a 31% reduction due to education support programme; and 31% reduction in the arm, where the girls received life skills training on gender and rights. One single intervention that demonstrated a 90% reduction in CM was only among very young girls aged 10–14 years [25]. The history of reductions in CM in Bangladesh indicates that it is easier to reduce very early CM compared to CM among older adolescents [26]. Currently, CM among older adolescents happens to be the bottleneck in the overall reduction of CM in Bangladesh any further [27].

The results of dose-response analyses show that the effect of TPI on all the secondary outcomes was in the expected direction, although only a few achieved statistical significance. It is interesting to observe that girls’ cohesion and collective efficacy increased significantly in TPP+, where more emphasis was put on these elements of the intervention.

Although no statistically significant main effect was achieved in TPI, the large within arm dose-response effect bears some important implications. First of all, to the best of our knowledge, this is the first study to present evidence on the dose-response effect of interventions on CM. The findings highlight that to achieve an effect on CM it is important for an intervention to offer a high number of sessions and ensure the participation of the girls in no less than 36–39 sessions.

Second, as pointed out by Kalamar [12], Lee-Rife et al. [13], and Cislaghi et al. [28] lack of understanding of social norms and how to change them effectively impedes the development of effective and sustainable CM prevention programmes. To date, only a very few studies have demonstrated an effect of social norms and empowerment programmes on reduction of CM [10,11]. Judging by the main effects, our study is no exception. However, the results of the dose-response analyses are encouraging. It is important to note that these effects were achieved among girls aged 16 to 18 years, an age group, where reducing CM is particularly challenging [27]. These effects were achieved despite a comprised implementation of the full package of TPI.

Also, we underline that TPI was effective in reducing CM despite implementation challenges due to an overlap with the COVID-19 pandemic. The originally planned TPI package could not be implemented due to the COVID-19 pandemic, a reduced version of the intervention was implemented instead. However, to minimise the loss, some sessions were conducted virtually over phone during lockdown. Due to technological difficulties virtual sessions could accommodate a small number of participants. Therefore, some sessions were merged for the sake of managing time. Thus, in total, 40 sessions were conducted with the girls instead of 45. In the TPP+ arm, out of 10 planned events for community mobilisation, only seven could be held. Thus, the differences between the two intervention arms were not as pronounced as planned. Moreover, since the same number of staff implemented the intervention in both arms, the added responsibilities of the TPP+ staff could have compromised the quality of intervention delivery. These factors may have been reflected in the results of the dose-response results, where TPP shows a larger effect than TPP+.

The literature suggests that during the pandemic, child marriage had actually escalated [9] due to: (1) financial problems and uncertainty; (2) school closure and uncertainty regarding education of the girls; and (3) a rise in the availability of desired grooms during the pandemic [29–31]. Thus, it is actually remarkable that TPI had a positive dose-response despite the pandemic.

The effect of the additional intervention elements in TPP+ (i.e. cross- gender and -generation dialogues, girls’ movement building and girl-led community sensitisation) on social norm around decision making regarding own marriage is an important achievement to be mentioned and to take note of in designing interventions.

The TPI and its evaluation suffer from some limitations. The study design allowed to assess the impact of TPI among study participants only and not in the wider community. However, in the intervention clusters most of the eligible adolescent girls were actually covered by the programme.

We calculated child marriage among girls aged 14–18 years participating in TPI. Thus, it remained unknown whether the girls who did not reach 18 years at endline will eventually get married before 18 or not. As in other studies, some unobserved differences among arms may have remained unadjusted in the regression analyses. Social desirability bias is often inherent to intervention evaluation studies. In order to minimise this bias, we allowed a 10-month freeze period between intervention completion and endline data collection. Since we conducted the study in the unique setting of a high prevalent district in northern Bangladesh, the findings may not be more widely generalisable. Despite these limitations this study addresses a critical gap in the literature presenting compelling evidence on effectiveness of a social norm-based intervention in reducing CM.

Our findings demand attention of the programme implementers, policy makers, and researchers devoted to elimination of child marriage. The results indicate that it is absolutely necessary to devise ways to promote session attendance of the girls. Further research is necessary to assess the full potential of the revised TPP and TPP+ interventions. Such studies need to be accompanied by cost analysis and sustainability assessment.

## Additional material


Online Supplementary Document
