# Supplementary material for: Effectiveness of combined interventions to empower girls and address social norms in reducing child marriage in a rural sub-district of Bangladesh: A Cluster Randomised Controlled Trial of the Tipping Point Initiative
Source: J Glob Health. 2024 Feb 23;14:04020. doi: 10.7189/jogh.14.04020 (PMC10903545; doi:10.7189/jogh.14.04020)
Supplement: Online Supplementary Document [file jogh-14-04020-s001.pdf]

Table S1. The baseline characteristics of the girls from different tertiles in both the TPP and TPP+ arms

Characteristics, % (n)	TPP (363)			P-value	TPP (366)			*P*-value
	Lowest (0-27)	Middle (28-34)	Highest (36-40)		Lowest (0-27)	Middle (28-34)	Highest (36-40)	
Age (years), % (n)								
12	23.53 (32)	21.01 (25)	22.22 (24)	0.96	21.31 (26)	23.02 (29)	17.80 (21)	0.74
13	28.64 (39)	33.61 (40)	29.63 (32)		22.95 (28)	27.78 (35)	23.73 (28)	
14	21.32 (29)	21.85 (26)	25.00 (27)		27.05 (33)	25.40 (32)	33.05 (39)	
15	25.47 (36)	23.53 (28)	23.15 (25)		28.69 (35)	23.81 (30)	25.42 (30)	
Wealth index, % (n)								
Lowest	22.06 (30)	17.65 (21)	18.52 (20)	0.83	18.85 (23)	26.19 (33)	16.95 (20)	0.21
Second	23.53 (32)	20.17 (24)	20.37 (22)		18.03 (22)	19.84 (25)	20.34 (24)	
Middle	15.44 (21)	13.45 (16)	12.96 (14)		27.87 (34)	17.46 (22)	24.58 (29)	
Fourth	17.65 (24)	26.89 (32)	24.07 (26)		16.39 (20)	22.22 (28)	26.27 (31)	
Highest	21.32 (29)	21.85 (26)	24.07 (26)		18.85 (23)	14.29 (18)	11.86 (14)	
Religion, % (n)								
Muslim	11.76 (16)	2.23 (3)	2.78 (3)	0.002	9.02 (11)	2.23 (3)	9.32 (9.32)	0.05
Non-Muslim	88.24 (120)	97.62 (116)	97.22 (105)		90.98 (111)	97.62 (123)	90.68 (107)	

Measurement of covariates

Individual-level variables

Girls' education: Girls' education was determined based on the number of completed years of schooling and categorized as follows: 0-5 years of education = 1, 6-7 years of education = 2, 8-10 years of education = 3, and 11 or more years of education = 4.

Girls’ religion: Girls’ religious affiliation was recoded as Muslim = 1 and Non-Muslim = 2.

Girls' ownership of assets: The ownership of assets among girls was assessed by asking the question, "Do you own any assets that could help you generate income?" with response options: Yes = 1, No = 0.

Group membership: Group membership was determined from the responses to the question, "Currently, do you regularly attend a group or organization meeting?" and coded as: Yes = 1, else = 0.

Household level variables

Wealth Index: The household wealth index was measured using a set of questions derived from the Bangladesh Demographic Health Survey (BDHS) 2017-18. A principal component analysis of household assets was applied to calculate the wealth index, which was further categorized into five groups: Poorest = 1, Poorer = 2, Middle = 3, Richer = 4, and Richest = 5.

Household Head's Education Level: The level of education of the household head was determined by the number of completed years of schooling and categorized as follows: no education = 0, 1-5 years of education = 1, 6-10 years of education = 2, and 11 or more years of education = 3.

Cluster/village level variables

Women's Education: The cluster level women's education was calculated as the average years of education completed among women in the corresponding clusters.

Religion: The cluster level religion was calculated as the proportion of Muslims in each cluster.

Community Member Characteristics

The age of community members was recorded in completed years at both baseline and endline. Their level of education was also categorized based on the number of completed years of schooling: no education = 0, 1-5 years of education = 1, 6-10 years of education = 2, and 11 or more years of education = 3. Marital status was categorized as: Currently married = 1, Divorced/Widowed/Widower/Separated = 2, and Never married = 3. Religious affiliation of community members was recoded as Muslim = 1 and Non-Muslim = 2.
